# Effect of maternal age on ATP content and distribution of mitochondria in bovine oocytes

**DOI:** 10.1371/journal.pone.0302444

**Published:** 2024-04-18

**Authors:** Dinesh Dadarwal, Luiz Pfeifer, Miriam Cervantes, Gregg P. Adams, Jaswant Singh

**Affiliations:** Department of Veterinary Biomedical Sciences, Western College of Veterinary Medicine, University of Saskatchewan, Saskatoon, SK Canada; Justus Liebig Universitat Giessen, GERMANY

## Abstract

Our objective was to understand how maternal age influences the mitochondrial population and ATP content of *in vivo* matured bovine oocytes. We hypothesized that *in vivo* matured oocytes from older cows would have altered mitochondrial number and distribution patterns and lower cytoplasmic ATP content compared to the oocytes obtained from younger cows. Follicles ≥5mm were ablated in old cows (13 to 22 yrs, Old Group, n = 7) and their younger daughters (4 to 10 years old, Young Group; n = 7) to induce the emergence of a new follicular wave. Cows were treated twice daily with eight doses of FSH starting 24 hr after ablation (Day 0, day of wave emergence). Prostaglandin F2alpha (PGF) was given on Days 3 and 3.5, LH on Day 4.5, and cumulus-oocyte-complexes were collected 18–20 hours post-LH by ultrasound-guided follicular aspiration. Oocytes were either processed for staining with MitoTracker Deep Red FM or for ATP assay. Stained oocytes were imaged with a Zeiss LSM 710 confocal microscope, and mitochondria were segmented in the oocyte volume sets using Imaris Pro 7.4. *In vivo* matured oocytes obtained from old cows were similar in morphological grades to those from young cows. However, the oocytes of COC from older cows had 23% less intracellular ATP (27.4±1.9 vs 35.7±2.2 pmol per oocyte, P = 0.01) than those of young cows. Furthermore, the average volume of individual mitochondria, indicated by the number of image voxels, was greater (P<0.05) in oocytes from older cows than in those from younger cows. Oocytes from older cows also tended to have a greater number of mitochondrial clusters (P = 0.06) and an increased number of clusters in the central region of the oocytes (P = 0.04) compared to those from younger cows. In conclusion, our study demonstrated that maternal age was associated with a decrease in the cytoplasmic ATP content of in vivo mature oocytes and an altered distribution of mitochondrial structures. These findings suggest that maternal age may negatively influence the developmental competence of oocytes from older cows.

## Introduction

A significant factor contributing to reduced fertility in women of advanced age is the decline in the developmental competence of oocytes [1,2]. In this context, a bovine model was developed and validated to study the influence of maternal age on various aspects of women’s reproduction [3–6]. Oocyte competence was found to be affected by the age of the cow; the number of oocytes from old cows that failed to undergo fertilization was twice that from younger cows [6]. Similar results were reported in subsequent in vitro fertilization (IVF) studies, with oocytes recovered from older cows exhibiting lower cleavage rates (58% versus 78%) and blastocyst production (14% versus 46%) than those from younger cows [7,8]. It is noteworthy that neither the survival of embryos nor calving rates differed when embryos from old and young cows were transferred to unrelated recipients. Disruptions in nuclear and cytoplasmic maturation, including lipid droplet number, mitochondrial number and their distribution, have been implicated in the decline in oocyte competence during maternal aging in women [9,10] and in follicular aging in the bovine model [11,12].

In a study of human oocytes, the estimated number of mitochondria increased tenfold from the number in primary oocytes of growing follicles to around 100,000 in ovulated mature oocytes [13]. Both mitochondrial number and distribution pattern have been associated with oocyte maturation and subsequent developmental competence [14–17]. The ATP content within the ooplasm is indicative of mitochondrial function and closely related to the organization of mitochondrial into clusters [16,18]. In mice, a positive correlation was observed between the ATP content of mature oocytes and subsequent embryonic development before genomic activation [19,20]. In cattle, a negative correlation was observed between mitochondrial numbers in *in vitro* matured oocytes and advancing age [7]. Recently, changes in mitochondrial population have been characterized *in vitro* compared to *in vivo* matured oocytes [21], but data regarding mitochondrial number, distribution, and ATP content of *in vivo* matured bovine oocytes in relation to the age of the cow have not been reported.

Using a bovine model, our aim was to investigate the impact of maternal age on mitochondrial numbers, distribution and ATP content of *in vivo* matured oocytes. We tested the hypotheses that *in vivo* matured oocytes from older cows exhibit an altered distribution pattern of mitochondria and a reduction in the ATP content compared to those from younger cows. Our results indicate that maternal age has a negative impact on ATP content and alters mitochondrial distribution, which may affect oocyte competence.

## Materials and methods

Hereford-cross cows used in the study were housed at the University of Saskatchewan’s Livestock and Forage Centre of Excellence, specifically at the Goodale Research Farm (located at 52°N and 106°W). Cows were accommodated in corrals and provided fed alfalfa hay and barley silage, along with free access to fresh water and mineral blocks. The experimental protocol received approval from the University Committee on Animal Care and was conducted in accordance with the guidelines of the Canadian Council on Animal Care (AUP#20080040). Unless otherwise specified, all chemicals were purchased from Sigma-Aldrich (Oakville, ON, Canada).

### Collection of cumulus-oocyte complexes (COC)

**Fig 1:**
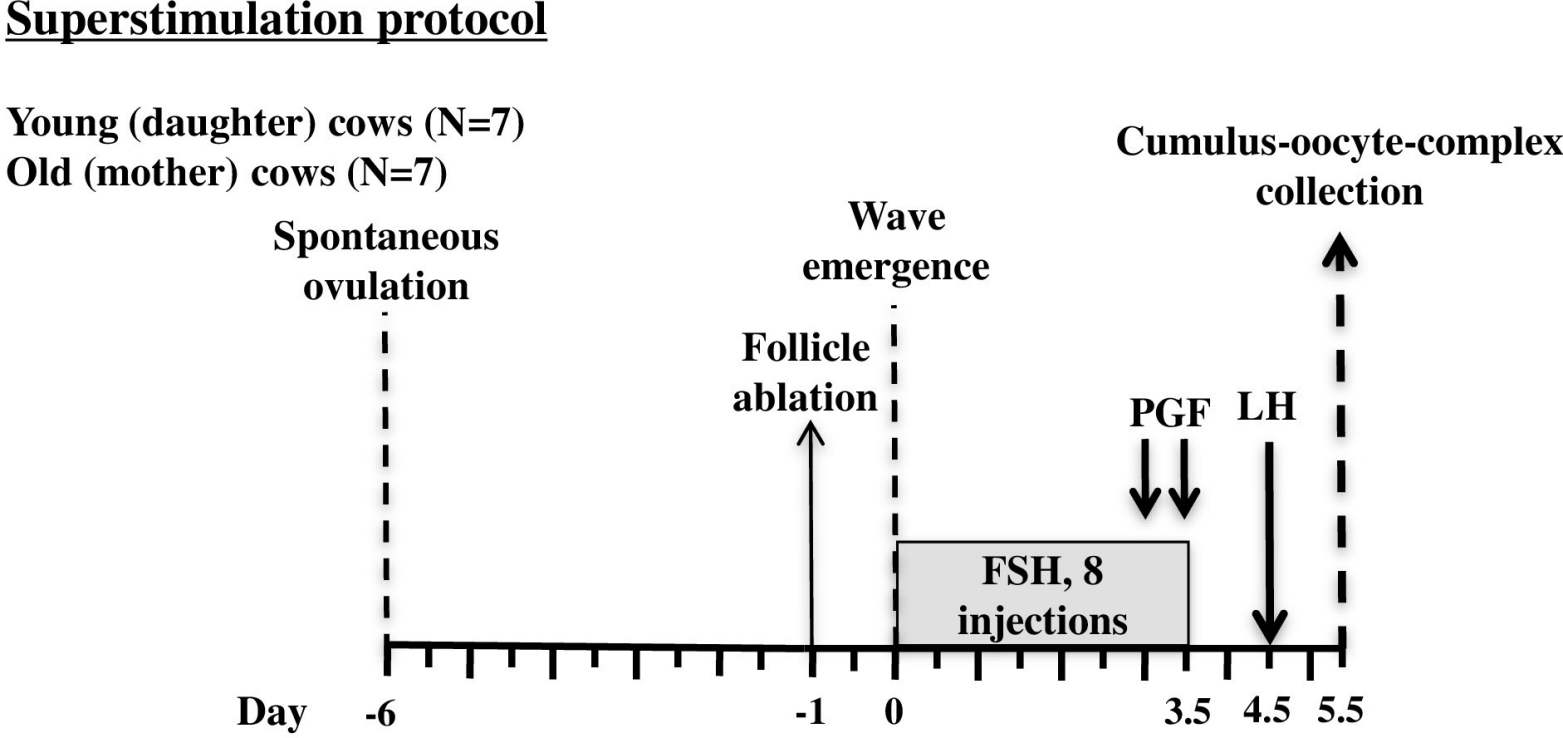
Protocol used to collect *in vivo*-matured cumulus-oocyte complexes (COC) from old cows (13–22 years of age; n = 5) and their younger daughters (7–10 years of age; n = 6) for the purposes of comparing ooplasmic mitochondria and ATP content. PGF = prostaglandin F2α; FSH = Folltropin; LH = Lutropin. FSH was administered at 12 h intervals starting on the day of wave emergence (Day 0). COC were collected by transvaginal ultrasound-guided aspiration of follicles ≥8 mm, 18–20 h after LH treatment.

COC were collected from two groups of cows; old cows aged 13 to 22 years (Old Group, n = 7) and their younger daughters aged 4 to 10 years (Young Group; n = 7) as depicted in Fig 1. Cows were subjected to daily transrectal ultrasonography to monitor ovulation. Six days after spontaneous ovulation, follicles measuring ≥5mm were ablated by ultrasound-guided follicle aspiration to induce the emergence of a new wave [22]. Starting a day later (i.e., expected day of follicle wave emergence, Day 0), cows were administered pFSH (6.25 mg NIH-FSH-PI units of Folltropin-V, Bioniche Animal Health, Belleville, ON, Canada) im at 12 h intervals for four days (a total of eight doses, with dose containing 50 mg FSH). A luteolytic dose of prostaglandin F_2a_ (PGF, 25 mg, Lutalyse, Pfizer Canada Inc., Montreal, QC, Canada) was administered im on Day 3 and 3.5 followed by pLH (12.5 mg, Lutropin-V, Bioniche Animal Health) im 24 hours after the last PGF treatment.

COC were collected 18–20 h after LH treatment using transvaginal ultrasound-guided follicle aspiration, employing a 7.5-MHz convex-array transducer (Aloka SSD-900, Hitachi-Aloka Medical Ltd, Tokyo, Japan) and a custom-made transducer handle, as previously described [23]. COC were identified by stereomicroscopy and subsequently washed 3 times in Dulbecco’s phosphate buffered saline (DPBS) containing 5% new-born calf serum (Invitrogen Inc., Burlington, ON, Canada). The COC were categorized as compact or expanded and graded from 1 to 4 based on the number of cumulus cell layers and the morphologic appearance of the ooplasm, as previously described [23]. To manage the number of COC on a given day, COC from each cow were pooled into three morphological categories: good (Grade 1), intermediate (Grade 2 and 3) and poor (Grade 4) [12]. The COC were denuded in a droplet of hyaluronidase solution (0.5% in Ca^2+^ and Mg^2+^ free DPBS, Invitrogen Inc.) and oocytes were either processed for mitochondrial staining or snap-frozen in liquid nitrogen (one oocyte per cryovial) for ATP assay.

### ATP assay

Oocytes were individually stored at -80°C in 50 μl of ATP sample buffer (99.0 mM NaCl, 3.1 mM KCl, 0.35 mM NaH_2_PO_4_, 21.6 mM Na-lactate, 10.0 mM HEPES, 2.0 mM CaCl_2_, 1.1 mM MgCl_2_, 25.0 mM NaHCO_3_, 1.0 mM Na-pyruvate, 0.1 mg/ml gentamicin, and 6.3 mg/ml BSA) [15]. A Bioluminescent Somatic Cell Assay Kit (Bioluminescent Somatic Cell Assay Kit, FL-ASC, Sigma-Aldrich, Oakville, ON, Canada) was used as per manufacturer’s instructions to assess the total ATP content per oocyte. A six-point standard curve (range 40 fmol to 125 pmol) was generated by including standards at the beginning and end in the assay. High, medium and low internal quality controls were created by processing three denuded oocytes and performing serial dilutions, as reported previously [12]. The coefficient of variation for high, medium and low internal quality controls were 6.8%, 5.6% and 3.3%, respectively.

### Processing of oocytes for mitochondrial staining and 3D segmentation

MitoTracker Deep Red FM (M22426, Molecular Probes, Invitrogen Inc., Burlington, ON, Canada) was used for staining mitochondria in denuded oocytes [12]. Stained oocytes were subsequently washed and fixed in 4% paraformaldehyde solution at room temperature for 1 hour. Following this, the oocytes were washed again and stored in DPBS in 1.5 ml centrifugation tubes and covered with aluminum foil to avoid photobleaching. A Zeiss LSM 710 confocal microscope (63x 1.4 oil objective lens, He Ne 633 laser, excitation: 645 nm, emission: 665–685 nm) was utilized for imaging and capturing 3D-volume sets. To do this, stained oocytes were mounted in Vectashield Mounting Medium containing DAPI (Vector Laboratories Inc., Burlingame, CA, USA) drops in glass bottom petri dishes (Catalogue # P35G-0-10-C, MatTek, Ashland, MA USA). A 20-micron thick 3D volume set was obtained with voxel sizes of 0.1μm x0.1μm x0.2μmresulting in (1352 x1352 x102 XYZ pixels),which was consistent with a previous report [12]. All image acquisition settings were maintained similar across oocytes, except for initial laser power, which varied to identify at least a few saturated pixels in the oocyte at a specified focal plane.

For the image processing, segmentation of the 3D volume sets, and generation of the quantitative data, we focused on the first 10-micron thickness of the 3-D images, specifically with the range of 20 to 30-microns from the ooplasm surface along the Z-direction of the 10-micron z-stacks. This section was used to identify and quantify both individual and clustered mitochondria and their distribution. The oocyte volume sets underwent 3-D deconvolution using Autoquant X2 software (Media Cybernetics, Rockville, MD, USA) [12,23]. After deconvolution, images were imported and processed further for segmentation of stained mitochondria in Imaris Pro 7.4 C1 package, which includes Imaris Track, Imaris Coloc, Imaris XT, Imaris Cell and Vantage modules (Bitplane AG, Badenerstrasse, Zürich, Switzerland). The identification of individual mitochondria, clusters and their distribution within oocytes using Imaris was carried out following a methodology similar to a previous study [12]. Inbuilt filters were employed to generate quantitative data for the number, mitochondrial staining intensity (representing mitochondrial activity), volume (average number of voxels), and distribution of mitochondrial structures within the peripheral and central regions of ooplasm.

Additionally, maximum intensity projection images (MIP) were generated from 2-micron z-stacks, focusing on the range of 28-30-microns from the ooplasm surface within the imaged volume set. These MIP images were used to determine the distribution pattern of mitochondrial intensities within the ooplasm. All MIP images were examined by a single observer (DD) who was blinded to the oocyte source. The observer characterized the images into distinct patterns, including scattered, clustered, peripherally clustered perivacuolar, or perivacuolar-clustered patterns [12].

### Statistical analyses

Statistical analyses were performed using the Statistical Analysis System software package (SAS 9.2; SAS Institute Inc., Cary, NC, USA) and probabilities ≤0.05 were considered statistically significant. The effect of maternal age on the proportion of COC grades and oocyte mitochondrial distribution patterns was assess through Fisher’s Exact test and Chi-square test using the Proc Frequency procedure. Proportional data for COC retrieved from follicles aspirated in the two age groups were compared using a non-parametric analysis of variance (Kruskal Wallis test). For mitochondrial number, average intensities and average number of voxels in oocytes from different age groups, a t-test was used to compare the data.

## Results

### COC collection efficiency and grades

COC were not obtained from two old cows and one daughter cow; hence the analyses are based on a sample of five old cows (aged 13 to 21 years) and six younger daughters (aged 4 to 10 years). The overall COC collection efficiency, calculated as the ratio of COC collected to aspirated follicles), was 178 out of 358 (49.5%), and this ratio did not show a significant difference (P = 0.80) between the two age groups (see Table 1). A small number of COC from the young group (5 COC) and the old group (3 COC) were lost before grading. The remaining COC were used for subsequent analyses. Overall, 90% of COC (154/170) had expanded cumulus cells, and the proportion of compact versus expanded COC did not differ (P = 0.40) between the age groups. Within the expanded COC, the proportion of different morphological grades also did not significant differences between the age groups (P = 0.12 for Good, P = 0.74 for Intermediate, P = 0.12 for Poor).

**Table 1:** Collection efficiency and morphologic grade of COC obtained from old cows (13–22 years of age; n = 5) and their younger daughters (7–10 years of age; n = 6) after ovarian superstimulation (FSH) and *in vivo* maturation (18–20 h after LH).

Endpoint*	Young cows	Old cows
Number of COC collected per cow (mean±SEM)	5.6+1.7	10.0+3.8
Total COC collected/total follicles aspirated (collection efficiency)	69/147 (46.9%)	109/212(51.4%)
Compact COC	8/64 (12.5%)	8/107 (9.2%)
Expanded COC	56/64 (87.5%)	99/107 (90.8%)
Expanded Good	25/56 (44.6%)	34/99 (34.3%)
Expanded Intermediate	21/56 (37.5%)	47/99 (47.5%)
Expanded Poor	10/56 (17.9%)	18/99 (18.2%)

* No significant differences between groups for above endpoints.

### Maternal aging: Total ATP content

**Fig 2:**
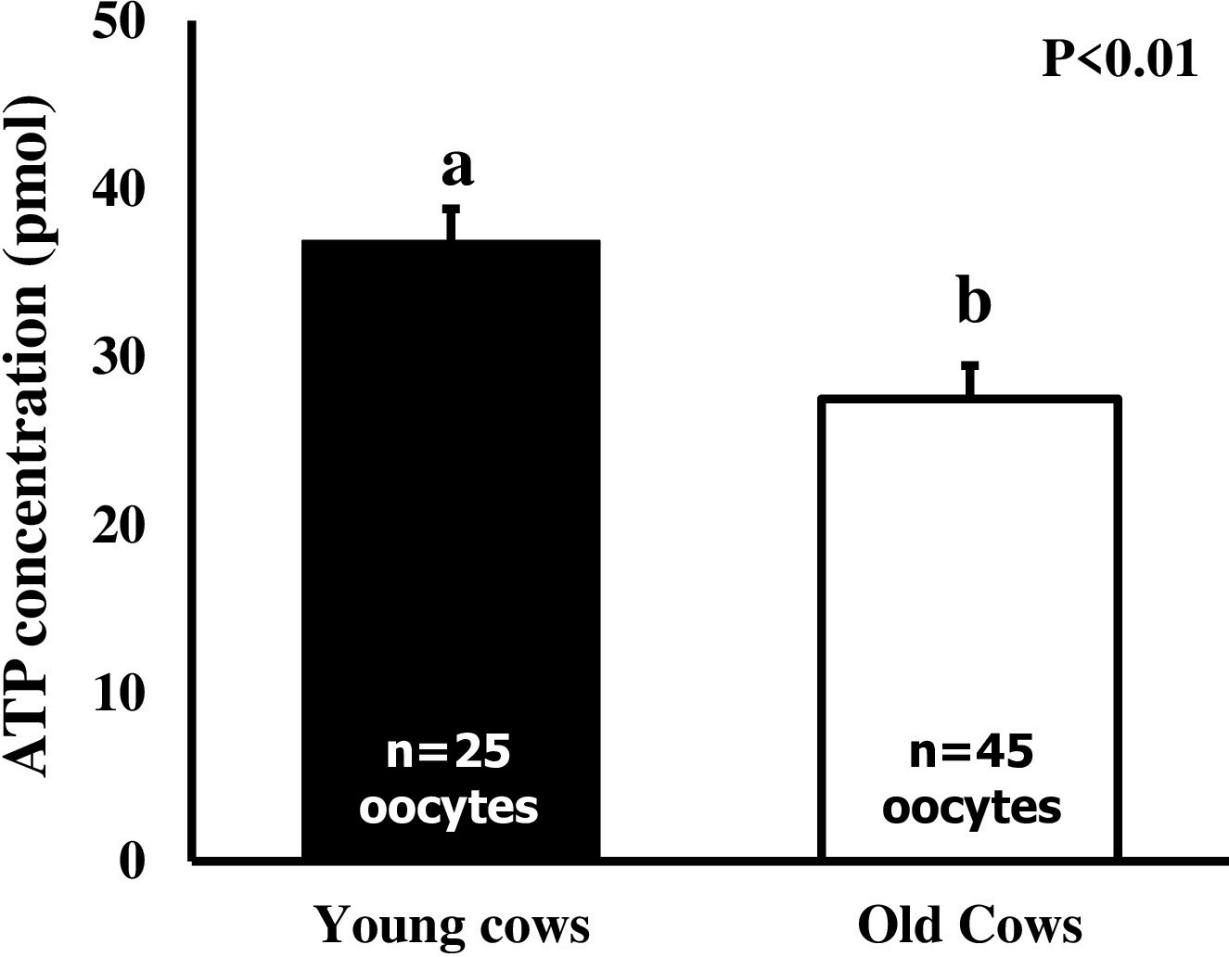
Total ATP content (mean±SEM) in the oocytes of old cows (13–22 years of age; n = 5) and their younger daughters (7–10 years of age; n = 6). Only oocytes from expanded COC (*in vivo* matured), regardless of morphological grade, were used. ^ab^Values with different superscripts are different (P<0.05).

A total of 29 oocytes from the young group and 48 from the old group were used to compare ATP content. Regardless of COC morphology (compact vs. expanded) and grade (1 to 4), the total ATP content in oocytes was 23% lower in the old group compared to the young group (27.4±1.9 pmol vs 35.7±2.2 pmol, P<0.01). While ATP content in oocytes from compact COC did not differ between the age groups, the ATP content of oocytes from expanded COC was lower (P<0.01) in old versus young cows (Fig 2). When expanded COC were compared irrespective of age group, poor-quality COC (Grade 4) exhibited lower ATP content than intermediate and good-quality COC (Grades 1–3; 10.1±5.3 pmol vs 31.2±1.6 pmol and 31.2±1.8 pmol, (P<0.01).

### Mitochondrial population

A fluorescent signal was not detected in 15 oocytes from the old group and 9 from the young group. Among the remaining oocytes, three COC in the old group and four in the young group were classified as compact (i.e., not matured) and not included for further analyses. Therefore, image analyses and group comparisons were made based on 42 oocytes from the old group and 20 oocytes from the young group.

The distribution of mitochondria within the ooplasm was categorized into five patterns based on visual examination of MIP images (as shown in Table 2). The proportion of oocytes with each distribution pattern did not differ (P = 0.18) between the two age groups. When combined across age groups, the majority of oocytes (77%; 46/59) displayed either a perivacuolar scatter or perivacuolar clustered mitochondrial pattern. The comparison of numbers and proportions of individual mitochondria and clusters (obtained from confocal volume sets) is provided in Table 3. The total number of mitochondrial structures and individual mitochondria were similar (P = 0.54) between age groups. The region-wise number of individual mitochondria also did not differ (P = 0.42) between the groups. Oocytes from older cows tended to have a higher proportion of individual mitochondria (P = 0.06) and their clusters (P = 0.06) than those of younger cows. The number of clusters in the central region of oocytes from old cows was higher than that of young cows (P = 0.04). In contrast, the number and proportion of clusters in the peripheral region of oocytes from young and old cows were not different (P = 0.12). The characteristics of mitochondrial population, size and staining intensities did not differ among the morphologic grades of COC when the age groups were combined.

**Table 2:** Mitochondrial distribution pattern in the oocytes of old cows (13–22 years of age; n = 5) and their younger daughters (7–10 years of age; n = 6) collected after ovarian superstimulation (FSH) and *in vivo* maturation (18–20 h after LH).

Distribution pattern*	Young cows	Old cows
Oocytes analyzed	20**	42
Uniformly scattered	2/20 (10%)	2/42 (4.8%)
Uniformly clustered	-	6/42 (14.3%)
Peripherally clustered	2/20 (10%)	1/42 (2.4%)
Perivacuolar scattered	4/20 (25%)	17/42 (40.5%)
Perivacuolar clustered	9/20 (45%)	16/42 (38.1%)

*No significant differences were observed.

** Three oocytes from young cows could not be categorize into any of the five specified distribution patterns.

**Table 3:** The number (mean±SEM) and proportion of mitochondrial structures within the oocytes of old cows (13–22 years of age; n = 5) and their younger daughters (7–10 years of age; n = 6) collected after ovarian superstimulation (FSH) and *in vivo* maturation (18–20 h after LH). The volume of the oocyte was estimated by ellipsoidal area of successive Z-stack images taken at 10-micron increments. Total structures represent individual mitochondria and mitochondrial clusters.

Endpoints	Young cows	Old cows
*Number of oocytes*	20	42
*Total structures*	1647±144	1774±126
*Individual mitochondria*: *Imaged volume*		
Average number	1493±135	1572±112
Proportion of total structures	0.90±0.01	0.88±0.01*
*Individual mitochondria*: *Peripheral region of imaged volume*		
Average number	715±48	683±40
Proportion of total individual mitochondria	0.52±0.04	0.46±0.01
*Individual mitochondria*: *Central region of imaged volume*		
Average number	778±109	889±79
Proportion of total individual mitochondria	0.48±0.04	0.54±0.01
*Mitochondrial clusters*: *Imaged volume*		
Average number	154±18	202±17*
Proportion of total structures	0.10±0.01	0.12±0.01*
*Mitochondrial clusters*: *Peripheral region of imaged volume*		
Average number	85±10	102±9
Proportion of total clusters	0.59±0.04	0.52±0.02
*Mitochondrial clusters*: *Central region of imaged volume*		
Average number	69±18 ^a^	100±9 ^b^
Proportion of total clusters	0.41±0.04	0.48±0.02

^ab^ Within rows, values with different superscripts are different (P<0.05).

* Within rows, values tended to differ (P>0.05 to <0.10).

### Average intensities and voxel numbers of mitochondria

**Fig 3:**
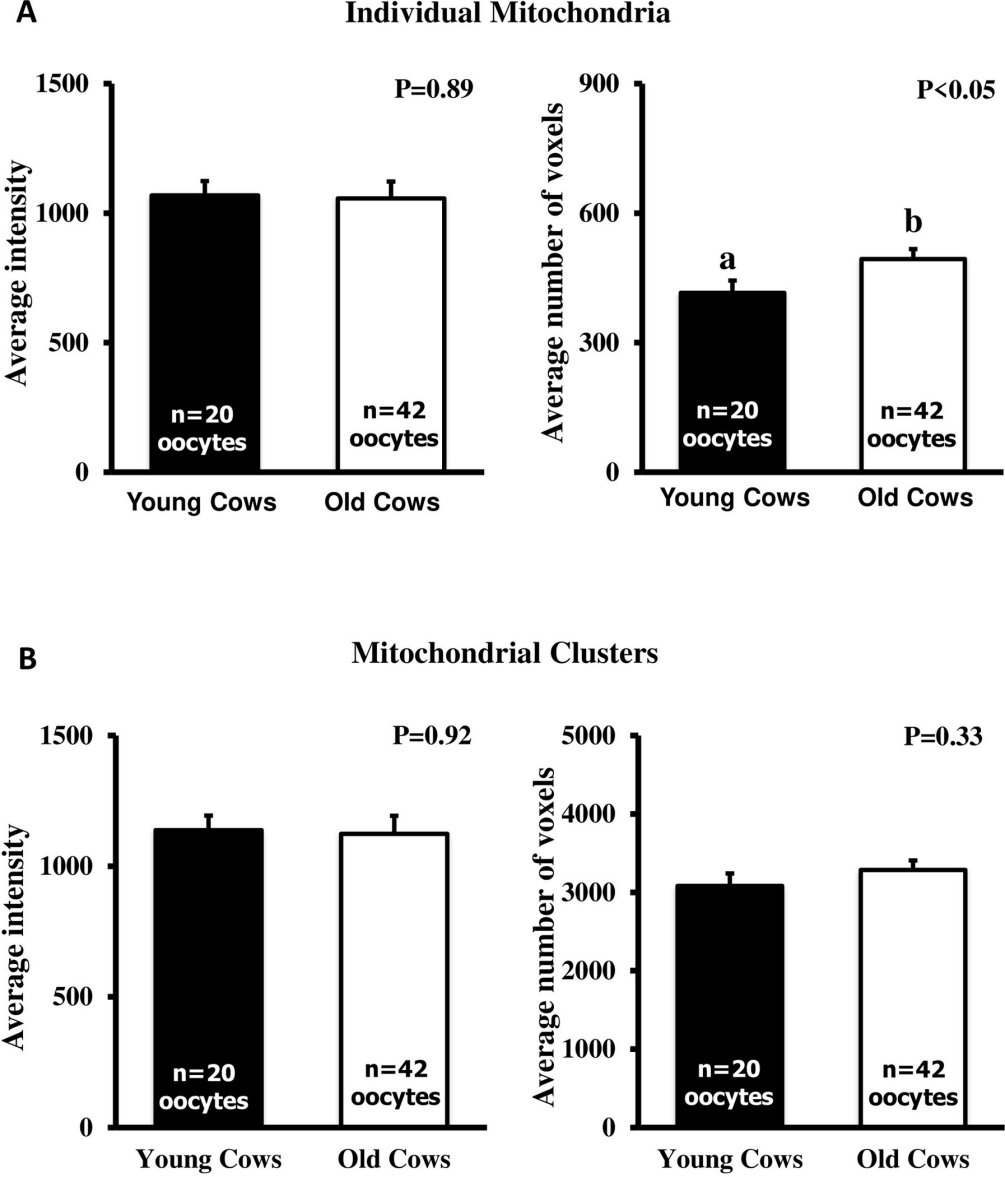
Intensity and number of voxels (mean±SEM) representing individual mitochondria and mitochondrial clusters in the oocytes of old cows (13–22 years of age; n = 5) and their younger daughters (7–10 years of age; n = 6). ^ab^ Values with different superscripts are different (P<0.05).

The mean staining intensities of individual mitochondria and clusters within the oocytes did not differ between age groups (Fig 3). However, the average volume of individual mitochondria, as indicated by the average number of voxels, was greater (P<0.05) in oocytes from old cows compared to those from young cows (Fig 3). Nevertheless, there was no significant difference (P = 0.33) between the two age groups in terms of the number of voxels representing mitochondrial clusters (P = 0.33).

## Discussion

Maternal aging did not have an impact on the morphological grades of *in vivo* matured oocytes obtained following superstimulation. However, *in vivo* matured oocytes from older cows exhibited lower ATP content and increased presence of mitochondrial clustering in the central part of the ooplasm compared to those from younger cows. These differences may provide an explanation for the observed loss of developmental competence of oocytes with advancing age in cattle, as noted in a previous study [6].

The relationship between maternal age and its impact on ovarian function have been systematically reviewed previously [24,25]. These studies have documented that cows aged 13–15 years exhibit an elevated level of FSH and reduced recruitment of follicles, mirroring ovarian changes similar to those reported in women approaching menopause [5]. Furthermore, maternal age is associated with a delayed preovulatory LH surge following estradiol treatment [4]. In addition, older cows tend to produce fewer large follicles after superstimulatory treatment compared to younger cows [3]. Moreover, oocytes from older cows exhibit significantly lower competence, resulting in only half as many embryos as those from their younger counterparts [6]. It was later documented that a suboptimal response to exogenous LH treatment in older cows, leading to delayed ovulation, is likely mediated by genes and pathways associated with delayed cellular differentiation, luteinization, and progesterone synthesis [26]. Despite these differences, it’s noteworthy that older cows had a similar or even a higher proportion of metaphase-II oocytes following superstimulation [27] Additionally, the proportions of ’morphologically’ good quality COCs obtained after 4-day superstimulation were not different between young and older cows in both the present study and a previous report [6].

The cytoplasmic ATP content of oocytes in the present study is higher than those reported in previous studies on bovine oocytes [14,15]. The difference may be attributed to the maturation status of the oocytes, as oocytes were matured *in vivo* in the present study, whereas they were matured *in vitro* in the above-cited studies. Notably, the *in vivo* matured oocytes from older cows had less intracellular ATP than those from younger cows. ATP is needed for energy-consuming processes such as cytoplasmic maturation and fertilization. While the oocytes of older cows may have sufficient ATP to undergo nuclear maturation, there may be insufficient ATP to complete fertilization and activate the embryonic genome. In a study of ATP content in human oocytes, in vitro maturation occurred over a wide range of ATP content, but the cohort of oocytes with >2pg/ml exhibited better fertilization rates and developmental competence [16]. In an early study involving *in vitro* maturation of bovine oocytes [7], authors reported higher ATP content in oocytes from old cows than young cows, but differences in the proportion of COC morphologic grades were not examined. We document herein that poor-quality oocytes had only 1/3^rd^ of the ATP content of intermediate or good-quality oocytes. The cohort of oocytes with more ATP content were correlated with greater developmental competence [15]. Moreover, differences in ATP content were not detected between oocytes obtained from slaughterhouse ovaries and those by ultrasound guided transvaginal follicular aspiration [28]. Although not assessed in the current study, the mitochondrial DNA copy number and ATP content were lower in cultured granulosa/cumulus cells obtained from older cows [29] and women [30] compared to those from respective younger counterparts.

Although oocytes from older cows had lower cytoplasmic ATP content, they tended to exhibit an increased number of mitochondrial clusters, primarily in the central region compared to oocytes from young cows. However, the intensity within these clusters, as well as the characteristics of individual mitochondria, did not differ significantly between the oocytes from the two groups. This observation contrasts the findings that clustering of mitochondria is related to ATP production in oocytes [16,18]. In our study, the number and proportion of individual mitochondria in the oocytes did not differ between the two age groups. In a study involving a different quantification method (mitochondrial DNA) [7], a decline in the number of mitochondria with advancing age was reported; however, there is a lack of correlation between the mitochondrial DNA copy numbers and ATP content of oocytes, presumably due to ATP generation by a subpopulation of mitochondria within an oocyte [31]. Given the dynamic nature of the mitochondrial population, capable of fusing, dividing, and moving to meet cellular energy demands, a crucial quality control mechanism like mitophagy becomes essential for rectifying damaged mitochondria. A recent study indicates that reduced mitophagy activators could lead to the accumulation of dysfunctional mitochondria in mouse oocytes, halting meiosis progression [32]. In that regard, dysfunctional mitochondria and energy metabolism have been associated with an imbalance of reactive oxygen species and their scavenging system in aged oocytes and granulosa cells, thereby resulting in age-related decline in fertility [33]. Perhaps central mitochondrial clustering is associated with altered oocyte metabolism due to maternal aging, resulting in lower ATP generation.

## Conclusions

In summary, maternal age was associated with a decrease in cytoplasmic ATP content of *in vivo* matured oocytes and an alteration in the distribution of clustered mitochondria. Central mitochondrial clustering and a decrease in cytoplasmic ATP may negatively influence the fertilization and developmental competence of oocytes from older cows.

## Supporting information

S1 Data Details of individual bovine oocytes collected from various animals enrolled in the study, depicting their oocyte quality, ATP content, and mitochondrial staining. (XLSX)
